# Mental Hospitals for Incurable Patients

**Published:** 1912-10-19

**Authors:** 


*To the Editor of* The Hospital.

### Mental Hospitals for Incurable Patients.

*To the Editor of* The Hospital.

Sir,—Will you allow me space in your interesting paper to make known a great want felt by a very large number of upper-class people, that of a mental hospital for incurable patients, who, though insane, still recognise their surroundings and fellow-sufferers? Mental hospitals under the London County Council arrange for these cases. Even when admitted as paupers the friends are allowed to clothe them, and they are kept separate from those who have had no education or refinement. In the county mental hospitals all are treated in the same way, and unfortunate gentlemen and ladies are dressed in the roughest clothes and associated with persons of the labouring class. Surely, even from a medical point of view, this must be wrong. It would cost the ratepayers much less if they allowed the friends to pay for clothes and laundress and provide a few extra comforts. There is one mental hospital endowed in England which takes incurable cases, but the management is so disgraceful that were I a rich man I would expose it at once. As it is, I cannot afford to fight, so have to keep silence. I feel sure this great want only needs making known to be remedied.—Yours faithfully,

